# Modified Castor Oil-Based Polyurethane Films with *Streptomyces* Extracts Presenting Anti-Methicillin-Resistant *Staphylococcus aureus* Activity

**DOI:** 10.3390/polym17172383

**Published:** 2025-08-31

**Authors:** Oscar T. Rodriguez, Luis E. Diaz, Manuel F. Valero

**Affiliations:** 1 Energy, Materials and Environment Group, Faculty of Engineering, Universidad de La Sabana, Chía 140013, Colombia; oscarrodga@unisabana.edu.co; 2 Master Program in Design and Management Process, Faculty of Engineering, Universidad de La Sabana, Chía 140013, Colombia; 3 Bioprospecting Research Group, Faculty of Engineering, Universidad de La Sabana, Chía 140013, Colombia; luis.diaz1@unisabana.edu.co

**Keywords:** polyurethanes, biomaterial, antibacterial, *Streptomyces*, cyclodextrins

## Abstract

Methicillin-resistant *S. aureus* is a problematic pathogen due to its high-risk infections and resistance mechanisms. To fight against this bacterium, novel antimicrobial sources and new delivery systems must be developed. Antimicrobial polyurethanes for developing biomaterials can function as preventive strategies. In this study, we explore the synthesis of partially renewable polyurethanes as biomaterial carriers of novel antimicrobials. An antibacterial extract from a *Streptomyces* sp. strain and its inclusion complexes with β-cyclodextrin, used as an additional protective approach, were incorporated into castor oil-based polyurethane films through bulk or surface loading. The inclusion complexes were characterized to confirm host–guest interactions. The films were characterized by FTIR, XRD spectra, surface SEM images, hydrophilicity, thermal stability, and mechanical performance. FTIR suggested successful polyurethane synthesis. The polymers were semicrystalline and thermally stable until 260 °C, and Tg ranged between −16.9 and −9 °C. Bulk modification decreased the mechanical performance of the films. Surface modification promoted good antibacterial performance but cytotoxic potential against HDFa cells. However, PU active films showed favorable properties and hemocompatibility, making them a promising alternative for applications such as short-term dressings, serving as an antimicrobial delivery system and a preventive strategy against methicillin-resistant *S. aureus*.

## 1. Introduction

Hospital-acquired infections represent a significant concern in the medical field. A substantial proportion of these infections is attributed to antimicrobial-resistant pathogens, so they have been mutually linked [1]. In 2019, 4.95 million deaths were attributed to antimicrobial resistance, with low- and middle-income countries being the most affected [2]. Methicillin-resistant *Staphylococcus aureus* (MRSA) is problematic due to its prevalence in nosocomial, severe acute, and chronic infections [3]. Its biofilm protection and genetic mechanisms promote multidrug resistance, reducing the number of effective antibacterial agents available, thereby being classified as a high-priority pathogen [2,4,5]. Infections range from skin lesions to infectious endocarditis, pneumonia, and sepsis [4,6].

Antibacterial biomaterials have emerged as a promising strategy for preventing, mitigating, or treating infections associated with medical devices [7]. Biomaterials serve as protective platforms and effective carriers for the delivery and controlled release of antibacterial agents [8]. As the pressure from resistant pathogens increases, advances in antibacterial biomaterials contribute to the optimization of antibiotics and infection prevention strategies [9,10]. These systems enable sustained antimicrobial release, allowing the employment of efficient amounts for local administration at higher concentrations to prevent and treat infections, providing better and safer results than systemic antibiotic therapies [11,12]. In this context, it is important to find adequate polymers and active substances that act as viable alternatives to continue the prevention and fight against methicillin-resistant *S. aureus*.

**Figure 1:**
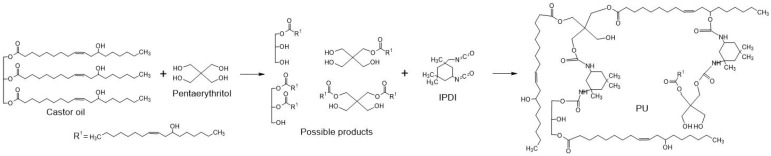
Reaction scheme of polyurethane from modified castor oil.

Among the polymers used to design biomaterials, polyurethanes have been broadly studied. Polyurethanes are highly studied due to their biocompatibility and versatility. They have been proposed for tissue engineering scaffolds, vascular grafts, wound dressings, and drug delivery [13,14,15]. Polyurethanes have shown outstanding physical and chemical properties. Synthesized through the polymerization of polyols and diisocyanates, they present a particular polymer backbone of soft and hard segments, respectively [16]. The structure of these segments provides special characteristics to polyurethanes: stiffness, elasticity, crystallinity, and thermal stability, among others [17,18]. Recently, research on PU synthesis has focused on the use of alternative monomers to petrochemical derivatives to improve the renewability of the process [19]. Castor oil is commonly employed as a polyol due to its favorable composition and natural hydroxyl groups [20]. However, it is poorly reactive, so it is usually chemically modified to increase the number and reactivity of hydroxyl groups [20,21]. The transesterification reaction with functional molecules, such as glycerol and pentaerythritol, increases the functionality of castor oil, producing polyols with primary and secondary hydroxyl groups suitable for high-performance polyurethanes (Figure 1) [20,22].

Several studies have investigated the loading of active agents into polyurethanes. For example, Shen et al. developed antimicrobial polyurethane films by blending natamycin with polyurethane and zein for blow-spinning applications [23]. Sehmi and coworkers impregnated glutaraldehyde into polyurethane biocidal materials [24], and Gulmez et al. incorporated drugs into castor oil-based polyurethanes through swelling processes [25]. In all cases, the active agents were incorporated into either the bulk or the surface of the developed materials. Antimicrobials can be incorporated into the bulk of the polymer or physically adsorbed by immersion, diffusion, or hydrophilic interactions in the surface [26].

Recently, the *Streptomyces* genus has proved to be a source of many antibacterial compounds. *Streptomyces* species secrete secondary metabolites from their metabolism, producing about 70% of the marketed antibiotics [27]. There are still around 3 thousand bioactive products described [28] with activity against bacteria, like *E. coli* and *S. aureus* [29]. The integration of polymers with *Streptomyces* extracts is only just being explored. Electrospun polyvinyl alcohol (PVA) nanofibers with *Streptomyces* extracts have demonstrated promising potential for wound dressing applications [30]. But the design of a biomaterial with bioactive substances must overcome, in many cases, limitations in compound stability and high toxicity. Thus, complexation of bioactive substances with cyclodextrins has been a useful strategy. As cyclodextrins act as hosts of active substances, they can improve their stability, solubility, and protection against oxidation and degradation [31,32]. Additionally, they can reduce the drawbacks of antibacterial agents and improve delivery systems [33].

**Figure 2:**
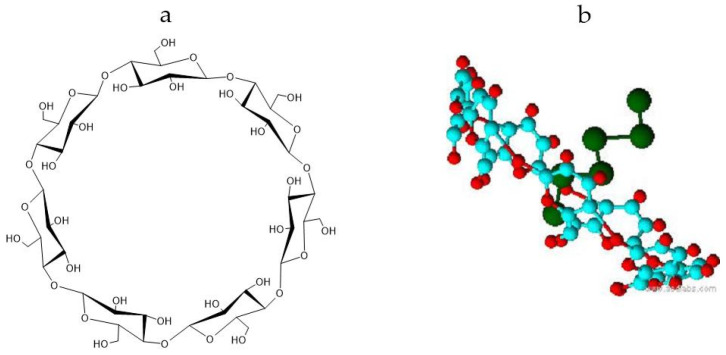
(**a**) β-Cyclodextrin and (**b**) inclusion complex structures.

Cyclodextrins are oligosaccharides with cyclic structures that resemble a torus (Figure 2). They have different polarities in the inner and external regions, a hydrophobic inner cavity, and a hydrophilic outer surface [32]. As a result, these molecules can host guest molecules, including polar and non-polar inorganic molecules and ions, forming inclusion complexes [34,35]. They are becoming a popular reagent because they can enhance bioavailability and membrane permeability and reduce the toxicity of active molecules [36,37].

Numerous studies have explored the incorporation of β-cyclodextrins into polyurethanes to enhance their properties and functionality. This modification has been shown to improve thermal stability and hydrophobicity [38], as well as degradability [39], and has been proposed to provide the polyurethane capture and release ability of active molecules [40]. The aim of this study is to explore the synthesis of partially renewable polyurethanes as biomaterial carriers of novel antimicrobials. Innovative loading strategies were studied, including bulk and surface modifications using the pristine extract from *Streptomyces* sp. and its inclusion complexes with β-cyclodextrin as an additional protective approach. The physicochemical, physico-mechanical, and thermal properties were investigated. In addition, the developed films were evaluated for biocompatibility in blood contact assays and for toxicity through cell viability tests according to international standards. Due to the advantages of the polyurethanes and the *Streptomyces* sp. extract, the materials have adequate performance in terms of hydrophilicity, thermal, and mechanical characteristics. These novel materials have proven to be antibacterial against the methicillin-resistant *S. aureus* (MRSA) pathogen and blood contact compatible. As a result, this configuration has the potential to be used in biomedical applications.

## 2. Materials and Methods

### 2.1. Materials

Castor oil (CO) was obtained from Químicos Campota y Cía Ltd.a (Bogota, Colombia). β-Cyclodextrin (CD) and isophorone diisocyanate (IPDI) were obtained from Sigma-Aldrich (Burlington, MA, USA). Pentaerythritol (PE) was obtained from Alfa aesar (Haverhill, MA, USA). All chemicals were analytically pure. *Streptomyces* sp. strains were obtained from the collection USAB-Sabana. The *Streptomyces* sp. strain was isolated from Arauca River sediments, obtained in Colombia, as methicillin-resistant *S. aureus* (ATCC BAA44). Phosphate-buffered saline (PBS) was obtained from RPI (Mount Prospect, IL, USA). 3-(4,5-dimethyl-2-thiazolyl)-2,5-diphenyl-2H-tetrazolium bromide (MTT), 2.5% trypsin (10×), and Dulbecco’s modified Eagle medium (DMEM, 1×) were obtained from Gibco/Invitrogen (Paisley, UK). Fetal bovine serum (FBS) was from Eurobio (Les Ulis, France). Triton^®^ X-100 was obtained from Thermo Scientific (Waltham, MA, USA). The CytoTox 96^®^ Non-Radioactive Cytotoxicity Assay kit was obtained from Promega (Woods Hollow Road, Madison, WI, USA).

### 2.2. Obtention of Streptomyces sp. Extract

The *Streptomyces* sp. strain was initially inoculated from a cryopreserved culture onto an ISP-2 agar plate (yeast extract 4 g/L, malt extract 10 g/L, dextrose 4 g/L, and agar 20 g/L) using the spread plate method. After a 7-day incubation at 30 °C, a plug (6 mm) of the culture was transferred into 3 mL of liquid ISP-2 medium and incubated at 19 °C with shaking at 130 rpm for another 7 days [41]. Subsequently, 1 mL of this culture was transferred into 9 mL of fresh ISP-2 and incubated again under the same conditions. This process was repeated to obtain a final culture volume of 600 mL. The biomass was then separated by centrifugation at 10,000× *g* rpm and 4 °C for 15 min. Then, a concentrate of the antimicrobial extract (EXT) was obtained by freeze-drying the supernatant [42].

### 2.3. Synthesis of Inclusion Complexes

Inclusion complexes (ICs) were obtained following the co-precipitation method with slight modifications [34]. A certain amount of CD was solubilized in 50 mL of 1 ethanol–2 water (*v*/*v*) solution at 50 °C for 2 h. Then, an amount of the EXT corresponding to a CD:EXT ratio of 4:1 was incorporated under constant stirring at 55 °C; agitation was maintained under stirring for another hour. The final solution was left to cool down at ambient temperature for 12 h and stored overnight at 4 °C to precipitate the complexes. The solid phase was recovered by vacuum filtration and then washed with distilled water. A physical mixture (PM) served as a control and was made by mixing solid phases CD:EXT (4:1) in a mortar.

### 2.4. IC Confirmation

#### 2.4.1. Thermal Analysis

The thermal transitions of the EXT, CD, PM, and ICs were investigated by differential scanning calorimetry on a DSC3+ (Mettler Toledo, Columbus, OH, USA). The sample was subjected to heat from 25 to 300 °C under N_2_ atmosphere (50 mL/min) with a 10 °C/min heating rate. The thermogravimetric analysis was evaluated on a TGA/DSC 1 (Mettler Toledo, Columbus, OH, USA) from 25 to 350 °C under a N_2_ atmosphere (50 mL/min) and a 10 °C/min heating rate [35].

#### 2.4.2. Furrier Infrared Spectroscopy (FTIR)

FTIR spectra were recorded in a range between 650 and 4000 cm^−1^ and resolution of 4 cm^−1^ using a Cary 630 FTIR Spectrometer (Agilent, Santa Clara, CA, USA).

#### 2.4.3. X-Ray Diffraction

The crystalline phases were investigated by XRD using an Aeris X-ray diffraction unit (Panalytical, Malvern, Worcestershire, UK) within a range of 5 to 20° (2θ). CuKα was used as the X-ray source with a scanning speed of 5 degrees/min and a voltage of 40 kV.

#### 2.4.4. Nuclear Magnetic Resonance (H-NMR)

The ^1^H-NMR spectra of the EXT, CD, PM, and ICs were assessed using a Bruker Avance NEO 400 MHz (Bruker, Billerica, MA, USA) at 25 °C and D_2_O as the solvent [43].

#### 2.4.5. Antibacterial Activity

IC antibacterial activity was investigated by the disc diffusion assay. A methicillin-resistant *S. aureus* (MRSA) suspension, 0.5 McFarlan, was inoculated into a trypticase soy agar plate (TSA) using the spread plate method. Then, 60 µL of a 0.5% IC solution was applied to 6 mm cellulose discs, which were placed onto the plate and incubated at 37 °C for 20–24 h. Finally, the antibacterial effect was analyzed by measuring the inhibition zone diameters (mm) employing a digital caliper.

### 2.5. Castor Oil Modification

Modified castor oil (MCO) was obtained by the transesterification reaction between castor oil and pentaerythritol [44]. In a three-neck reactor equipped with a thermometer, reflux condenser, nitrogen atmosphere, and agitation, CO was heated for 10 min at 120 °C under stirring. The temperature was increased to 210 °C, and 1.3% mol PE and 0.05% lead oxide, as a catalyst, were incorporated. The stirring and temperature were maintained for 2 h. Then, MCO was allowed to settle down to recuperate from the decanted catalyst.

### 2.6. Synthesis of Polyurethane Films

The polyurethane films were synthesized using the prepolymer method, with the polymerization reaction of IPDI with MCO as a source of isocyanate and polyol, respectively [45]. The reaction was carried out for 5 min in a reactor at 60 °C, with a magnetic stirrer at 300 rpm and reagents at a NCO/OH molar ratio of 1, forming the prepolymer solution of PU. For PUCD, a homogenized powder of β-cyclodextrin was incorporated in the prepolymer solution and allowed to mix for another 5 min. The prepolymer solutions were cast on individual glass plates and spread out with a film applicator at 0.5 mm using an Elcometer 3580 Casting Knife Film Applicator (Elcometer Ltd., Manchester, UK). Then, the films were transferred to a 110 °C oven for 12 h for the curing process. Finally, the films were peeled off and stored until further analysis. The films were labeled as PU and PUCD.

For the bulk-modified polyurethanes in each case, homogenized powder of the EXT, the CD-Ext mixture, and the IC were incorporated on the prepolymer stage as described above. The films were labeled as PU_ExtB, PUCD_ExtB, and PU_ICB.

For the surface-modified polyurethanes, the PU and PUCD films were soaked in 0.5% aqueous Ext solution, while the PU film was soaked in 0.5% aqueous IC solution for 24 h. Afterwards, the polyurethane films were left to air dry. The films were labeled as PU_ExtS, PUCD_ExtS, and PU_ICS.

### 2.7. Film Characterization

#### 2.7.1. Structural, Morphological, and Crystalline Characterization

To confirm structures and syntheses, FTIR of the PU-based films was recorded on an ATR-FTIR spectrometer Cary 630 FTIR Spectrometer (Agilent, Santa Clara, CA, USA) in a range between 650 and 4000 cm^−1^ and resolution 4 cm^−1^ [46].

Surface morphology was obtained using scanning electron microscopy (SEM). Images were taken at 6 kV voltage acceleration, 1.0 kx magnification, using a Vega 4 (Tescan, Brno, Czech Republic) and gold metallized coating.

The crystalline structure of the materials was described by XRD in an Aeris X-ray diffraction unit (Panalytical, Malvern, Worcestershire, UK). CuKα was used as the X-ray source with a scanning speed of 5 degrees/min, a voltage of 40 kV, and a measuring range of 10–80° (2θ). Circle samples were employed for the analysis.

#### 2.7.2. Physicochemical Analysis

The affinity of the materials to water, hydrophilicity or hydrophobicity, was identified by the sessile droplet contact angle test using a MobileDrop GH11 (Krüss, Germany). In this assay, a 10 ± 2 µL drop of water at 20 °C was applied to the samples using a micrometer syringe. The shape of the droplets was analyzed at different locations to take measurements [47].

The swelling or water uptake was assessed by the gravimetric method following the standard ASTM D570, where squared samples of 50 × 50 mm were weighed and immersed in 45 mL of distilled water for 24 h. After this time, the films were gently wiped and weighed again. The percentage of water absorption was calculated using Equation (1), where *m_i_* is the initial mass of the dry sample and *m_f_* is the final mass after swelling [39,48].

(1) $$\%swelling=\frac{m_{f}-m_{i}}{m_{i}}\times 100$$

#### 2.7.3. Thermal Analysis

Thermal transitions of the materials were obtained using DSC on a DSC3+ (Mettler Toledo, Columbus, OH, USA) in programs from −70 to 150 °C in a 20 mL/min nitrogen atmosphere (20 mL/min) and sample weights of 10 mg [45]. Thermal stability was evaluated by thermogravimetric analysis on a TGA/DSC 1 (Mettler Toledo, Columbus, OH, USA) in a program consisting of a 20 °C/min heating rate, a 25–600 °C temperature range, nitrogen atmosphere (100 mL/min), and 15 mg of the tested samples [39,49].

#### 2.7.4. Mechanical Analysis

The mechanical profile was determined following the standard ASTM D882-18 for thin plastic films [50] with dimensions 20 mm × 5 mm × 0.25 mm (length × width × thickness) using a DMA 850 (TA Instruments, New Castle, DE, USA) with a crosshead speed of 10 mm/min and a 5 kN load cell.

### 2.8. Film Biological Activity

#### 2.8.1. In Vitro Antibacterial Activity

The antibacterial assessment was performed by the disc diffusion assay. Discs of each PU-based material, 6 mm in diameter, were UV-sterilized for 5 min. Then, the methicillin-resistant *S. aureus* (MRSA) suspension, 0.5 McFarlan, was inoculated into a TSA agar plate (trypticase soy agar) using the spread plate method. Then, the materials were placed on the plate and incubated at 37 °C for 20–24 h. Finally, the antibacterial effect was analyzed by measuring the inhibition zone diameters (mm) employing a digital caliper.

#### 2.8.2. Human Whole Blood Collection

The process of human blood collection was approved by the Ethics Committee in Act 68 of 18 May 2018, project identification code ING-2018-2019. All subjects gave their informed consent for inclusion before participating in this study, and the collection was performed under aseptic conditions. Fresh blood was collected in blood collection tubes with 3.2% sodium citrate to make anticoagulated blood.

#### 2.8.3. Hemolysis

To assess the hemocompatibility of the materials, hemolytic activity was investigated [51]. PU-based disc samples (6 mm) were sterilized by UV radiation for 1 h and stabilized by a 24 h PBS incubation. Then, the anticoagulated blood was stabilized by making a solution of 5% anticoagulated blood in sodium chloride (0.9%). Each sample was placed in a 2 mL microcentrifuge tube, and 500 µL of the blood was exposed to each sample. A negative control was prepared with 200 µL anticoagulated blood in 10 mL NaCl solution, and a positive control was prepared with 200 µL anticoagulated blood in 10 mL distilled water. The assay was incubated for 2 h at 37 °C. After this time, the tubes were centrifuged at 1000× *g* for 10 min, and 100 µL of each supernatant was placed in a 96-well plate to read the absorbance at 570 nm using an ELx800 absorbance microplate reader (BioTek instruments, Winooski, VT, USA). The percentage of hemolysis was evaluated according to Equation (2).

(2) $$\%hemolysis=\frac{{ABS}_{sample}-{ABS}_{negative\;control}}{{ABS}_{positive\;control}-{ABS}_{negative\;control}}\times 100$$

#### 2.8.4. Blood Clotting Time

The hemostatic performance of the developed materials was obtained by the blood clotting time assay [51]. PU-based disc samples (6 mm) were sterilized and stabilized as described above. Each sample was placed in a 2 mL microcentrifuge tube. Then, anticoagulated blood was recalcified (0.0091 M CaCl_2_) with 0.1 M CaCl_2_ to initiate the clotting cascade. Carefully, 50 µL of the recalcified blood was deposited on each sample. The assay was incubated at ambient temperature (19 °C) for 10, 20, and 40 min independently. After that, 1.5 mL of distilled water was added and incubated for another 5 min. Then, 200 µL of the assay was placed on a 96-well plate to read the absorbance at 570 nm using an ELx800 absorbance microplate reader.

#### 2.8.5. Platelet Adhesion

Human platelet adhesion was investigated using platelet-rich plasma (PrP) obtained after centrifuging anticoagulated blood for 10 min at 120× *g*. PU-based disc samples (6 mm) were sterilized and stabilized as described above. The samples were placed in 2 mL plates with 200 µL PrP and incubated at 37 °C for 1 h. After this time, the samples were rinsed with PBS to eliminate non-adhered platelets. The samples with platelets adhered were exposed to 200 µL 1% triton and incubated at 37 °C for 1 h to lyse the cells. The adhered platelets were quantified by the LDH quantification assay using the CytoTox 96^®^ Non-Radioactive Cytotoxicity Assay kit (Woods Hollow Road, Madison, WI, USA), following the manufacturer’s instructions [51].

#### 2.8.6. Cell Culture

HDFa cells were incubated with 5% CO_2_ at 37 °C in a T-75 cell culture flask with DMEM medium supplemented with 10% FBS and 1% penicillin–streptomycin. The medium for the cells was changed every 2 days until 100% confluence was reached. After that, the cells were trypsinized (trypsin–EDTA) [20].

#### 2.8.7. Cell Viability

HDFa cell viability in the PU-based films was investigated following the MTT method [20,44]. Cell suspensions at 4 × 10^4^ density were seeded in 96-well plates and incubated at 37 °C and 5% CO_2_ for 24 h. After that time, PU-based samples of 5 mm (previously sterilized in UV for 1 h and stabilized in supplemented medium for 30 min) were placed in contact with the cells and incubated for 24 h at 37 °C and 5% CO_2_. After finishing the exposure time, the samples and medium were removed, and 100 µL MTT reactant (12 mM in PBS) was added. The assay was incubated at 37 °C for 4 h. The supernatant was removed, and 100 µL DMSO was added and incubated again for 15 min at 37 °C. Finally, the absorbance was read at 570 nm in an ELx800 absorbance microplate reader. Cell viability was evaluated using Equation (3); untreated cells were used for the control (100%).

(3) $$\%Cell\;viability=\frac{{ABS}_{sample}}{{ABS}_{control}}\times 100$$

### 2.9. Statistical Analysis

Each assay was analyzed using parametric and nonparametric statistics at a significance criterion of *p* < 0.05. The results are expressed as mean ± standard deviation.

## 3. Results

### 3.1. Inclusion Complex Confirmation

**Figure 3:**
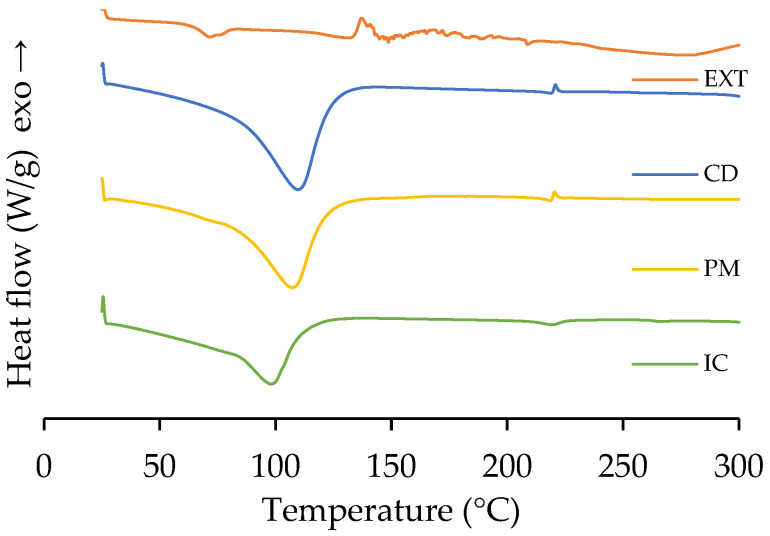
Calorimetric DSC curves of the extract (EXT), β-cyclodextrin (CD), the physical mixture (PM), and the inclusion complex (IC).

The modification of β-cyclodextrin toward the inclusion complex could be explored by DSC analysis (Figure 3). The pristine EXT presents a thermal transition that starts at 55 °C, corresponding to the melting process, and then thermal degradation reactions begin at 135 °C. Pure CD presents a thermal transition at 110 °C, corresponding to the dehydration process of residual water molecules in its hydrophobic cavity [52]. The PM shows a slight shift to the left (107 °C) due to the possible weak interactions with the microbial extract. However, it behaves identically to CD. Conversely, the IC shows a considerable decrease in intensity of the endothermic peak and a shift towards 98 °C. The active molecules could replace water in the hydrophobic cavities of β-cyclodextrin. The change in the thermal transition of the IC is in accordance with the results of inclusion complexes reported in the literature [31,34,35].

**Figure 4:**
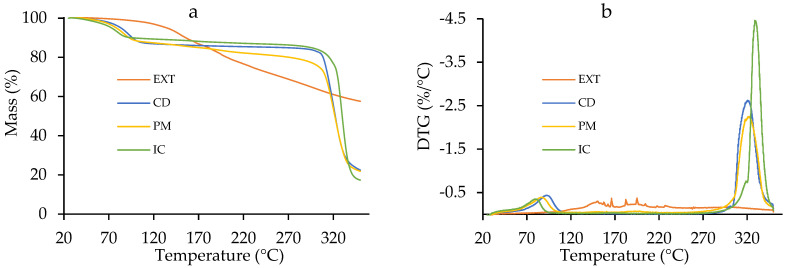
(**a**) Thermogravimetric TGA and (**b**) derivative DTG curves of the extract (EXT), β-cyclodextrin (CD), the physical mixture (PM), and the inclusion complex (IC).

The TGA analysis shows that the crude EXT maintains a constant weight for the first 60 °C, after which the compounds volatilize (39% of the mass), leaving behind residual ashes (Figure 4a). CD presents a step starting at 25 °C and ending at 110 °C, corresponding to bounded water elimination (~12%), and achieves decomposition at 320 °C (Figure 4a,b). The PM exhibits a first mass loss of ~12% between 25 °C and 105 °C and finally reaches degradation at 320 °C, which is in accordance with CD and its relative concentration in the mixture. In addition, it presents a uniform mass loss of ~13% between 105 °C and 260 °C that can be attributed to the volatile compounds of the extracts. The IC shifted the initial step starting at 25 °C and ending at 92 °C (Figure 4b), attributed to residual water (~8%). It is hypothesized that the difference in water loss between CD and the IC is due to the replacement of structural water by new host molecules and that the observed loss represents superficial water [53]. Also, thermal degradability occurs in two stages at 318 °C and at 330 °C due to complexed molecule release and β-cyclodextrin decomposition, respectively. This suggests the formation of an inclusion complex [54].

**Figure 5:**
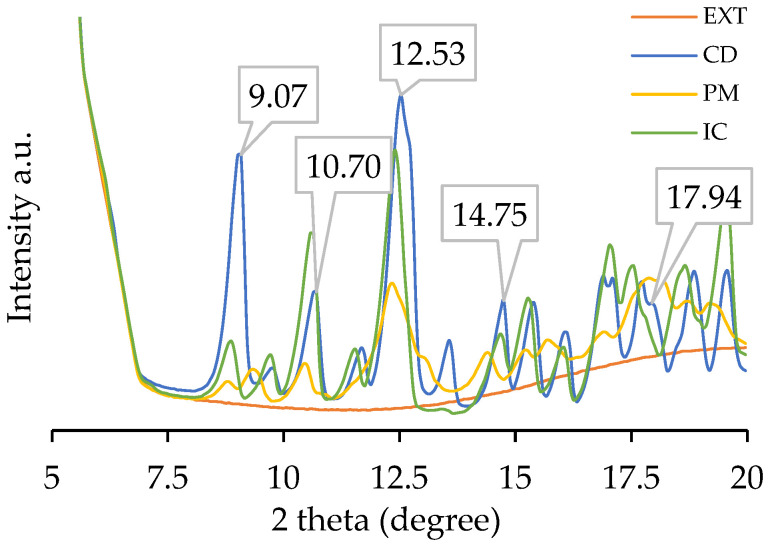
XRD patterns of the extract (EXT), β-cyclodextrin (CD), the physical mixture (PM), and the inclusion complex (IC).

The XRD pattern confirms the phase changes of CD after complexation with the extracts (Figure 5). First, the EXT suggests a completely amorphous sample. CD presents a crystalline structure, and the pattern has characteristic peaks at 9.07, 10.70, 12.53, 13.57, 14.75, and 17.94 (2θ). The mixture with extracts (PM) smooths the pattern, indicating interference of the extracts with the crystallinity of CD. Finally, the IC has a crystalline structure like CD. The pattern loses the 13° peak, and the 12° peak slightly shifts to a lower position. This can be attributed to complexed molecules, which can give an amorphous structure to the molecule [55]. The loss and appearance of diffraction peaks suggest the development of new crystalline structures and the complexation of molecules [36,56].

The ^1^H-NMR spectra (supporting material) help us determine whether or not there are active host–guest interactions between CD and EXT molecules, for which disturbances in the environment shift the chemical signals of protons (Table 1), mainly because the IC is formed by weak interactions [55]. The spectrum of CD has the peaks of H-2 and H-4 (3.56–3.51 ppm), H-5, H-6, and H-3 (3.79–3.89 ppm), and H-1 (5.00 ppm) [57,58]. The molecules that interact with the internal cavity of CD cause low-frequency shifts in the H-5 and H-3 protons [59]. Both the PM and IC caused modifications to the internal region of CD, suggesting host–guest interactions. In contrast, in the IC, an intense proton signal appeared at 1.1040 ppm, which was absent in CD and indistinguishable in the PM. This can be due to an upfield chemical shift in the proton (H_ext_) in the EXT. This suggests that a molecule belonging to the extracts is completely included in CD, forming an inclusion complex, even though the chemical shifts were not intense. Betlejewska-Kielak et al. obtained no considerable shifts in protons because of the large excess of β-cyclodextrin in relation to ketoprofen when preparing an inclusion complex by the co-precipitation method [60].

**Figure 6:**
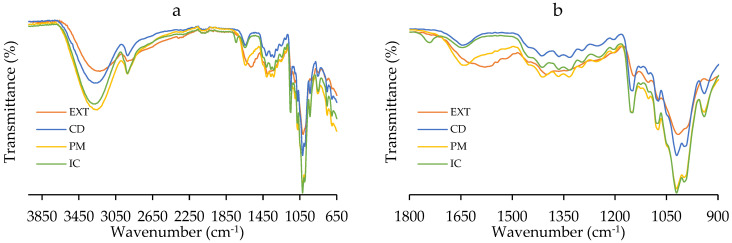
(**a**) FTIR spectra and (**b**) 1800 cm^−1^ to 900 cm^−1^ zoom of the extract (EXT), β-cyclodextrin (CD), the physical mixture (PM), and the inclusion complex (IC).

The FTIR spectra of the samples presented in Figure 6a,b support the findings of the NMR. CD has a broad at 3300 cm^−1^ peak (O-H stretching), 2925 cm^−1^ (C-H stretching), 1645 cm^−1^ (H-O-H bending), 1152 cm^−1^ (C-O-C asymmetric stretching), and 1019 cm^−1^ (C-O-C symmetric stretching) [57]. The EXT spectrum also has O-H and C-H stretching peaks and two broad bands at 1570 cm^−1^, for possible N-H or C = C bending, 1400 cm^−1^ (C-H bending), and 1010 cm^−1^ (C-O stretching), and resembles that of aminoglycoside molecules, like streptothricin [61]. Both the PM and the IC present similar spectra to CD, but the PM spectrum is considerably different, presenting the broad 1570 cm^−1^ peak of the EXT (Figure 6b). Instead, in the IC, the 1570 cm^−1^ broad band of the EXT is reduced, and the fingerprint behaves with modified intensity compared to CD. These results are consistent with the work reported by Branković et al., suggesting host–guest molecular interactions [36].

**Figure 7:**
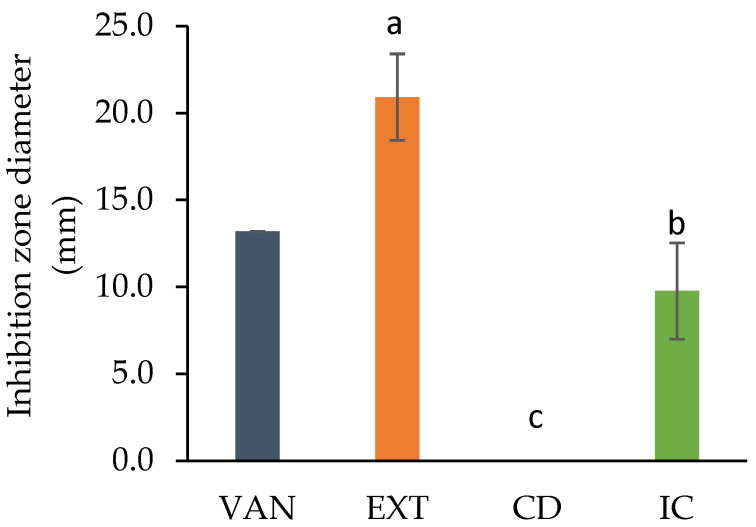
Antibacterial activity against methicillin-resistant *S. aureus* of vancomycin (VAN), the extract (EXT), β-cyclodextrin (CD), and the inclusion complex (IC). Samples with the same letter do not have a significant difference (Tukey test).

The resistance of the methicillin-resistant *S. aureus* (MRSA) pathogen was tested against vancomycin (VAN) discs containing 5 µg. The antibiotic has a power of inhibition of 13.2 mm (Figure 7). The EXT demonstrates a remarkable 158.47% higher efficacy compared to VAN in inhibiting bacterial growth. The *Streptomyces* sp. strain under investigation has been identified as a prolific producer of a complex mixture of novel bioactive substances. Some of the bioactive metabolites, like streptothricin, interfere with the synthesis of proteins possessing antibacterial properties [61]. The absence of an inhibition zone in CD (0 mm) indicates that it has no activity against the pathogen. The observed activity in the IC comes exclusively from the active metabolites, not from cyclodextrin. Thus, the sustained antibacterial activity of the IC indicates the successful retention of bioactive molecules within the complex.

### 3.2. PU Film Structure, Morphology, and Crystalline Characterization

**Figure 8:**
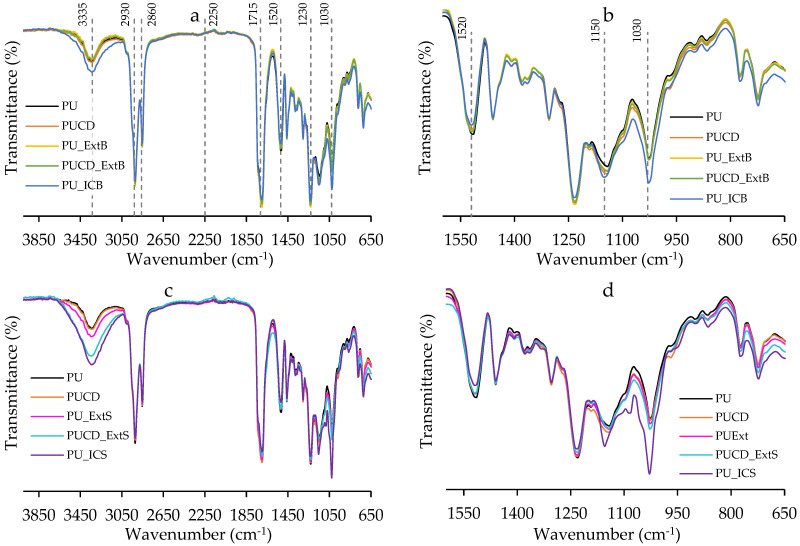
FTIR spectra of PU-based films. (**a**) PU bulk-modified derivative spectrum, (**b**) 1600 cm^−1^ to 650 cm^−1^ zoom, (**c**) PU surface-modified derivatives spectrum, and (**d**) 1600 cm^−1^ to 650 cm^−1^ zoom.

The characterization of the chemical structures in the PU-based films was achieved by FTIR spectroscopy, as shown in Figure 8. The spectrum of the PU films (Figure 8a) shows expected urethane peaks at 3335 cm^−1^, 2934 cm^−1^, 2861 cm^−1^, and 1715 cm^−1^, which are -NH stretching, -CH_2_ symmetric vibration, -CH_2_ asymmetric vibration, and C=O groups, respectively. The absence of the isocyanate peak at 2250 cm^−1^ (-NCO) confirms total consumption of the IPDI reactant and an adequate polymerization reaction, resulting in a polyurethane-type polymer. Additionally, peaks at 1520 cm^−1^, representing -NH (amide II) bending in the -HNCOO- group, 1470–1362 cm^−1^, representing CH_2_ bending, and 1230 cm^−1^, representing -NH (amide III) stretching, are typical of castor oil polyurethanes [39,62,63,64].

The spectrum of the PU derivatives shows the same shape as the pristine PU infrared spectrum, with a slight modification of the intensity of the 1520 cm^−1^ (-NH), 1150 cm^−1^, and 1030 cm^−1^ (-CO) peaks (Figure 8b–d). Peaks of the dextrin structure occur at 1150 cm^−1^, 1092 cm^−1^, and 1020 cm^−1^ (Figure 6), which can explain the shift in the signals, indicating that pristine β-cyclodextrin and inclusion complexes were included in the films and may form weak hydrogen bonds of hydroxyl groups [39,40].

The spectrum of PU_ICB, PU_ExtS, PUCD_ExtS, and PU_ICS showed 3350 cm^−1^ peak intensity modification, which can be related to an increased availability of OH groups and novel hydrogen bonds created in the films (Figure 8a–c) [65]. After deeper analysis, the 1715 cm^−1^ (C=O) signal decreased. Rahayu et al. reported a decrease in signal intensity after diffusing lavender essential oil into a polyurethane matrix, attributed to hydrogen bond interactions between the essential oil molecules and the urethane groups, which altered the vibration frequency [65]. Comparing PU_ICS with PU_ICB, we can suggest that surface modification increases the availability of complexed molecules, as the 1030 cm^−1^ peak has the highest intensity.

**Figure 9:**
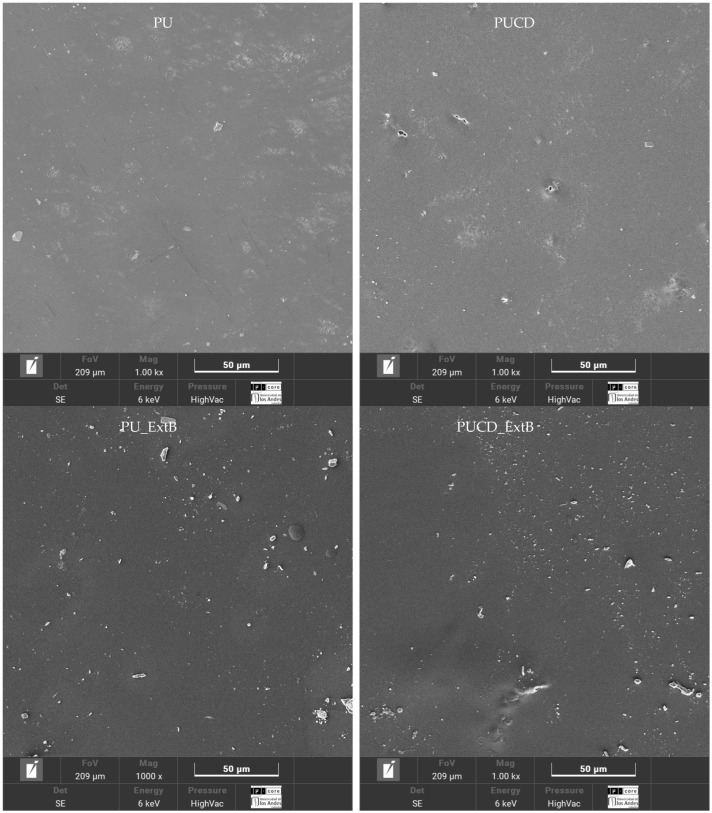

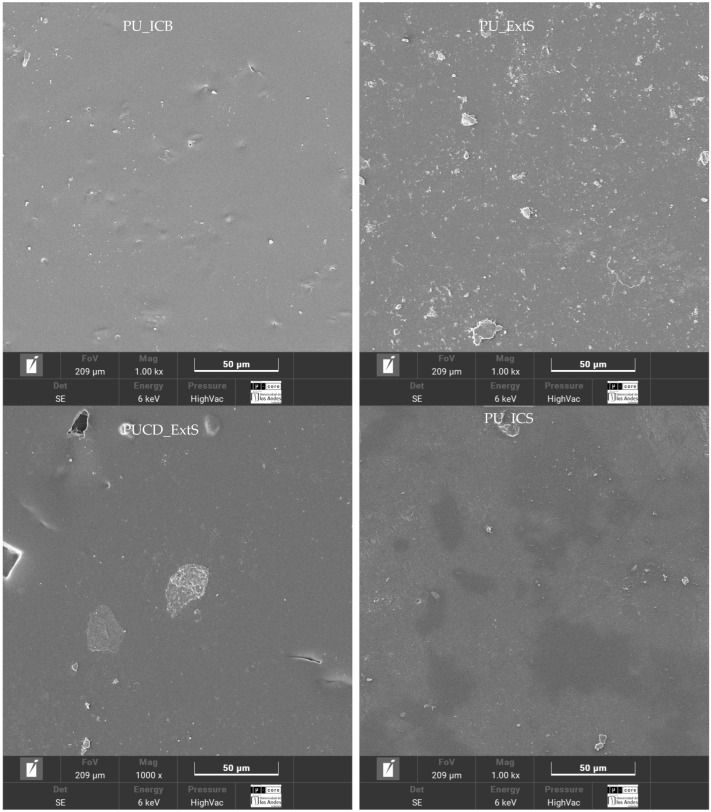
Surface SEM micrographs of the developed films, 1.0 kx.

Figure 9 shows the morphology of the synthesized polyurethane films. The PU surface presents a relatively smooth morphology with adhesive properties, as microscopic atmospheric particles with a diffuse shape can be observed in it. All the films present the same characteristics. While the bulk-modified polyurethanes (PU_ExtS and PUCD_ExtS) present well-shaped, defined particles, attributed to the content of solid extracts, the higher definition of the shape makes the extract particles different from adhered atmospheric particles. The inclusion of β-cyclodextrin induced the appearance of pores. The surface-modified polyurethanes present interesting surface characteristics. PU_ExtS have a broad number of shapes attributed to atmospheric particles, extract interactions, and phase separation in the polymer matrix. In PUCD_ExtS, an increased shape of pores and dense zones of β-cyclodextrin arrangements are observed. Gulmez et al. observed similar behavior after loading drugs in polyurethanes from PEG and CO by the swelling method [25]. In contrast, PU_ICS shows opaque areas, which can be attributed to zones with smoother surfaces and variable thicknesses.

**Figure 10:**
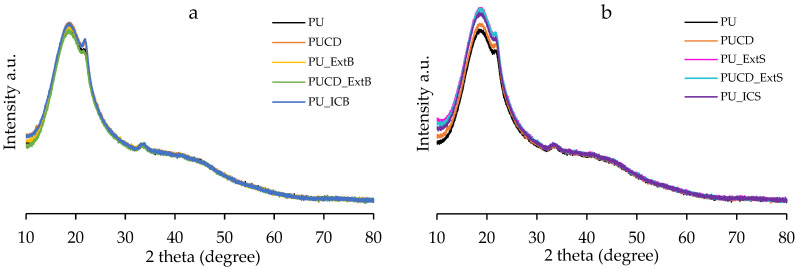
XRD patterns of PU-based films. (**a**) PU bulk-modified derivative patterns and (**b**) PU surface-modified derivative patterns.

The XRD presented in Figure 10 shows that the PU-based films are mainly semicrystalline, and their pattern presents a broad peak near 20° at 2θ. Microphase separation of soft and hard segments led to amorphous and crystalline domains [66]. Also, the low crystalline structure of the polyurethane films resulted from their crosslinked and highly branched nets [67]. The inclusion of β-cyclodextrin did not modify the film crystallinity, as the diffraction of PUCD remains almost the same as PU. The bulk-modified PU films have the same amorphous arrangement (Figure 10a). The representative diffraction peaks of the pure β-cyclodextrin and inclusion complex were reduced in both the surface- and bulk-modified polyurethanes (Figure 10a,b). However, for the surface-modified PU films, the intensity of the broad peak increased. Less intense diffraction peaks are related to low microphase separations and a highly crosslinked structure [66,67]. As shown in Figure 10b, the surface modification may rearrange the domains and slightly increase phase separation and crystallinity.

### 3.3. Physicochemical Analysis

The surface hydrophobicity and wettability of the polyurethane films were assessed by droplet contact angle measurements (Table 2). The PU-based films have low hydrophobicity, as the values of the contact angle remained below 90° [20]. The PU film presents the least hydrophilic nature, with a contact angle of 88.97 ± 3.71°. Instead, inclusion of β-cyclodextrin decreases the contact angle in the PUCD film, but not significantly. One possible explanation may be the increase in hydrophilic sites resulting from the incorporation of β-cyclodextrin [39]. The multiple hydroxyl groups of β-cyclodextrin can be exposed on the polymer surface; this increases the interaction between water and hydrophilicity [62,68]. Bulk modification presented the same tendency. Employing extracts decreases the contact angle, maybe due to phase separation and their soluble nature.

Surface modification with extracts has a significant effect on the wettability of polyurethanes. The contact angle decreased by almost 26° by means of surface modification with the extracts. This indicates that a surface modification was achieved. The rearrangement and surface-concentrated extracts may be the reasons for the increased hydrophilicity. Pardini and coworkers found a significant decrease in the contact angle after loading PU-based films by the immersion method, attributing the result to the surface distribution of the drug molecules, which were hydrophilic [69]. Instead, the bulk and surface modification with the inclusion complex were not significantly different from PU and PUCD.

Moreover, the water absorption analysis reveals that after 24 h of soaking, the PU film reached 0.42 ± 0.031% water absorption. Bhosale and coworkers found a similar trend in their castor oil-based polyurethane films [70]. This can be explained by the hydrophobic nature of castor oil-based soft segments [71]. Swelling is a phenomenon influenced by various factors, including molecular conformation. A previous investigation reported a low swelling capacity of IPDI-based polyurethanes [72]. Additionally, the crosslink density dictates swelling performance. Zhang et al. found that a lower crosslink density increased the water absorption of polyurethane films [73]. Most of the modifications did not affect water uptake significantly. But only the absorption of the PUCD_ExtS film was significantly decreased. It is possible that a rearrangement occurred and that void spaces were occupied by the extract molecules, as reported in a previous study, leading to a decrease in swelling compared to the non-loaded samples [25].

### 3.4. Thermal Analysis

**Figure 11:**
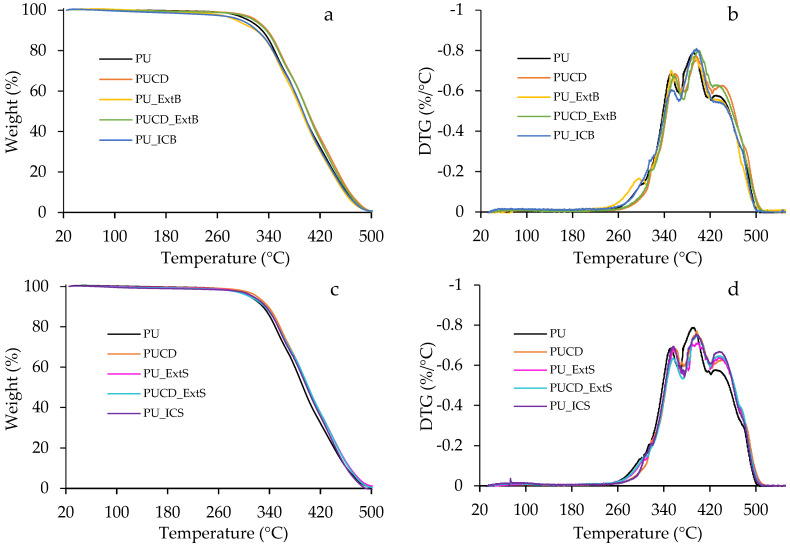
Thermogravimetry analysis: (**a**) TGA of bulk-modified PU films, (**b**) derivative curves of bulk-modified PU films, (**c**) TGA of surface-modified PU films, and (**d**) derivative curves of surface-modified PU films.

Figure 11 shows the thermogravimetric analysis of the PU-based films. All the films exhibit a similar behavior, being thermally stable until ~260 °C. In general, three steps are slightly observed during the TGA test (Figure 11a–c), which are confirmed in the DTG curve (Figure 11b–d). The first mass loss step, between 260 and 340 °C, can be attributed to hard segment and urethane linkage breaks [74,75]. Then, the second step between 340 and 390 °C is attributed to soft segment degradation and the remaining urethane decomposition [74,75,76]. The final step, between 380 and 440 °C, corresponds to final thermo-oxidative degradation and decomposition of soft segments [75,76]. The addition of β-cyclodextrin, extracts, and inclusion complexes by bulk and surface modification did not have a considerable effect on the thermal stability and degradation of polyurethane films. For PU_ExtB, PU_ExtS, and PUCD_ExtS, a short step occurs in the range of 296–310 °C (Table 2). This phenomenon can be due to the thermal instability of the extracts in the polymer system. However, the degradation temperatures are too high and do not limit the possible medical application of the material.

**Figure 12:**
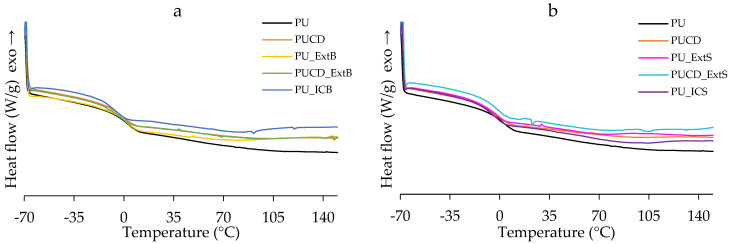
DSC thermograms of (**a**) bulk-modified PU films, and (**b**) surface-modified PU films.

Furthermore, the DSC thermograms (Figure 12) show the thermal transitions of the films. In accordance with the TGA results, all the films present similar performances. The glass transition temperature (Tg) changed from one design to another (Table 2). But, regarding PU_ExtB, all the modifications considerably decreased Tg. Decreased values of Tg indicate less restriction in chain mobility [77]. For the case of bulk-modified polymers, β-cyclodextrin and inclusion complexes may develop incompatibilities, disrupting polymer chain packaging. In the case of surface modification, the soaking may rearrange the soft and hard domains, resulting in less ordered homogeneous structures. However, the values of Tg are below −9 °C. This means that the biomaterials are viscoelastic at ambient and physiological temperature (19–37 °C). In addition, we can affirm that the polyurethanes have a homogenous backbone, as only one Tg transition is observed [74].

### 3.5. Mechanical Analysis

Polyurethane mechanical characteristics vary with respect to the configurations of soft and hard segments, the structures of raw materials, and hydroxyl groups of polyols [74]. The MCO- and IPDI-based polyurethane films exhibited outstanding stress and strain behaviors (Table 2). Their performance also correlates with the previously observed trend in Tg and the semicrystalline nature of the films. Mechanical performance is attributed to the unique equilibria between crystalline and amorphous domains. The inclusion of β-cyclodextrin to the polyurethane matrix did not significantly affect polymer performance (*p* > 0.05). However, it can support hydrogen bonds, which can explain the slight increase in tensile strength compared to base PU [78]. Also, between the materials with the same type of modification, no statistical differences were observed. While the maximum stress of the modified materials did not differ significantly from PU and PUCD, the stress was significantly lower (*p* < 0.05) in the bulk-modified PU_ExtB and PUCD_ExtB compared to the surface-modified PUCD_ExtS and PU_ICS. Similarly, PU_ExtS and PUCD_ExtS differ in strain compared to PU. These results can be explained by the fact that extracts act as particles that interfere with the polymer interpenetrated net, after which failure points appear. In addition to this, the surface modification seemed to generally increase the stress of the materials and reduce the strain. An explanation may be that soaked polyurethane undergoes a reorganization in the polymer matrix and the allocation of new substances, as shown in XRD. Previous studies have reported that hydrophobic polyurethanes undergo aggregation after being soaked in water [79]. In this way, surface-modified polyurethanes slightly increased the tensile strength while decreasing the strain, and then became brittle.

### 3.6. Antibacterial Activity

**Figure 13:**
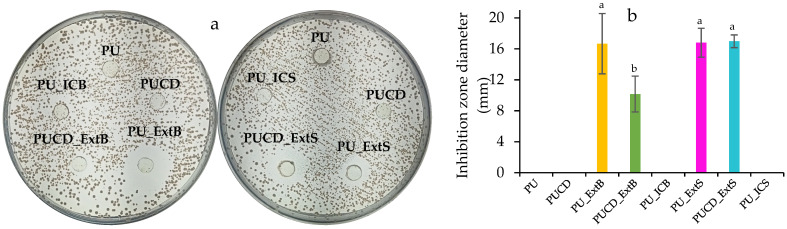
Zone of inhibition against methicillin-resistant *S. aureus* (MRSA). (**a**) Disc diffusion assays. (**b**) Inhibition zones.

The ability to fight against pathogenic bacteria can be seen in Figure 13. The clear zone that surrounds the polymer discs following 24 h of tests is evidence of antibacterial activity against methicillin-resistant *S. aureus* (MRSA). As expected, the polyurethane blanks, PU and PUCD, did not present any activity. Additionally, the inclusion-complex-loaded polyurethanes did not exhibit bacterial inhibition either. Inhibition was found in the polymers loaded with the extracts. No differences were observed between the surface-modified ones and PU_ExtB (~16 mm); this can be explained by the fact that the polymers have the same concentration of extracts. While PUCD_ExtB has a lower concentration of extracts in relation to polymer mass, the inhibition zone was found to be the smallest (~10 mm). In this way, bulk-modified polyurethane activity is sensitive to the relative proportion of extracts. Also, the inclusion of β-cyclodextrin did not significantly improve the grafting of bioactive molecules, as no statistical differences between PU_ExtS and PUCD_ExtS were observed. Khan et al. demonstrated a similar performance of polyurethanes loaded with β-cyclodextrin, which could absorb and release antimicrobial Ag NPs and inhibit *S. aureus* (<20 mm) [80]. In summary, castor oil-based polyurethanes can load and release antibiotics. This performance is particularly useful for wound healing, mitigating the associated complications of bacterial infections, especially in chronic wounds [81].

### 3.7. Hemolysis

When designing biomaterials for medical applications, the hemolysis assay is highly representative of biocompatibility [82,83]. The hemolysis assay suggests the interaction between materials and red blood cells (RBCs) and the harmful effect on cell integrity, after which hemoglobin is released [84]. The results of the hemolysis assay are displayed in Table 3. The polyurethane films can be considered not hemolytic, as the values remained below 2% [85]. The inclusion of β-cyclodextrin did not exhibit a significant effect on the hemolytic behavior of the polyurethane polymers. The same occurred with the inclusion of complexed molecules. No significant differences were observed between the modified materials and their respective blanks: PU and PUCD. Despite this, the most hemolytic polymer was found in PUCD_ExtB, which is not significantly different from the others except for PU_ExtS, which is the least hemolytic. The developed films are safe to use as blood contact biomaterials.

### 3.8. Blood Clotting Time

The blood clotting assays allowed us to identify if the polyurethane materials intervene in clot formation. Whole blood coagulates only after 20 min; almost all red cells are involved in clot formation (Table 3). Before this time, non-clotted red cells are lysed by water. Absorbance is higher when fewer cells participate in clot formation. The polyurethane films significantly accelerated clot formation after 10 min (*p* < 0.05). Coagulation within 10 min of contact with the polyurethane films was comparable to that observed at 40 min for the blood alone. No statistically significant differences were detected among the treatment groups. Accelerated blood clot formation indicates the hemostatic performance of the developed materials. For the healing of acute injuries, hemostatic wound dressings would be desired, as they can stop bleeding [86].

### 3.9. Platelet Adhesion

Platelet activation and agglomeration is one of the first stages in the coagulation cascade and activation of the intrinsic pathway. A low interaction between platelets and materials indicates good hemocompatibility [87]. The LDH released from lysed platelets increases medium absorbance in the LDH assay at 490 nm and is a way to measure the number of platelets adhered to materials (Table 3). The obtained materials did not exhibit platelet adhesion, as the values of absorbance were similar to the absorbance of the negative control (without PRP). Although the surface-modified polyurethanes tended to increase platelet adhesion, no significant differences were observed between the films. The results of the platelet adhesion test may indicate that clotting can be driven by other kinds of interactions, such as flocculation and surface charges [88]. Therefore, the polyurethane films developed in this study can be considered non-hemolytic, hemostatic, and hemocompatible, making them suitable for use in wound-dressing applications.

### 3.10. Cell Viability

The cytotoxicity of PU-based materials is a characteristic that needs to be evaluated to assess their proper biomedical application (Table 3). In the MTT assay, viable cells are quantified by evaluating their metabolic activity. Only the pristine PU had insignificant cytotoxicity; meanwhile, the inclusion of β-cyclodextrin significantly affected polyurethane biocompatibility (*p* < 0.05). Previous reports have obtained similar results for their β-cyclodextrin-loaded polyurethanes [40]. Despite that, PUCD can still be considered as having no cytotoxic potential (>70%) according to the ISO 10993-5 standard [89]. Both bulk and surface modifications resulted in less biocompatible polymers. The incorporation of the extracts led to the most significant viability decrease. And the surface-modified polyurethanes had the overall most cytotoxic performance. Previous studies have obtained similar cytotoxic effects from bioactive metabolites produced from *Streptomyces* isolates, and the toxic activity has been related to harmful metabolites (polyketides and alkaloids), which can disrupt cellular functions and membranes, causing damage and apoptosis [42,90]. As expected, based on the results of antibacterial activity, the inclusion complexes in polyurethane films (PU_ICB and PU_ICS) seemed to reduce the harmful effect of the extracts. Β-cyclodextrin masked the harmful activity of the extracts. Although antimicrobial polyurethanes have a slight cytotoxic potential, they are better candidates for short-term wound dressing or non-implantable biomedical devices.

## 4. Conclusions

In this study, anti-methicillin-resistant *S. aureus* (MRSA) and hemocompatible castor oil-based polyurethanes were synthesized. Two approaches for the development were analyzed, namely, bulk and surface modification with antimicrobial extracts from *Streptomyces* sp. cultures. In addition, inclusion complexes (β-cyclodextrin–extracts) were tested for possible beneficial effects on the overall performance of the films. Inclusion complexes were successfully obtained, according to deeper characterization. In the same way, the successful synthesis of polyurethanes was confirmed. In the case of the bulk-modified films, the additives (inclusion complexes, β-cyclodextrin, or extracts) acted as fillers carried by the polyurethane polymer matrix. Some weak bonds and crosslinking effects were identified in both the bulk and surface modifications. All the polyurethane films presented outstanding thermal, anti-methicillin-resistant *S. aureus* (MRSA), and hemocompatibility performance. The bulk-modified films presented mechanical property drawbacks. The surface-modified films presented the worst cytocompatibility. Regarding the employment of pristine extracts and inclusion complexes, the complexes hindered the antibacterial and cytotoxic activity of the extracts in the polymer system. Hence, it is important to consider novel ways to develop polyurethanes with both anti-methicillin-resistant *S. aureus* (MRSA) activity and non-cytotoxicity. However, the performance of the antibacterial films remained adequate for short-term biomedical applications, supporting the healing process in the prevention of infections from methicillin-resistant *S. aureus* (MRSA) pathogens.

## Figures and Tables

**Table 1:** H-NMR chemical shift (δ) of protons in β-cyclodextrin (CD), the physical mixture (PM), and the inclusion complex (IC).

H	CD (ppm)	PM (ppm)	IC (ppm)	EXT (ppm)	Δδ_PM_ (ppm)	Δδ_IC_ (ppm)
H_1_	5.0003	5.0003	4.9977	---	0.0000	0.0026
H_2_	3.5660	3.5655	3.5623	---	0.0005	0.0037
H_3_	3.8997	3.8956	3.8947	---	0.0041	0.0050
H_4_	3.5168	3.5181	3.5147	---	−0.0013	0.0021
H_5_	3.7900	3.7814	3.7822	---	0.0086	0.0078
H_6_	3.8101	3.8077	3.8064	---	0.0024	0.0037
H_EXT_	---	0.9630	1.1040	0.9629	−0.1411	−0.0001

**Table 2:** Contact angle, water absorption, thermal steps, and mechanical properties of the polyurethane films.

PU	Contact Angle (°) *^1^	Water Uptake (%) **^2^	Decomposition Steps, DTG Peaks (°C)	Tg (°C)	Stress (MPa) **^2^	Strain (%) **^2^
PU	88.96 ± 3.71 ^a^	0.42 ± 0.031 ^a^	T1. 351 T2. 389 T3. 430	−9	2.68 ± 0.39 ^ab^	257.17 ± 24.88 ^a^
PUCD	84.49 ± 4.29 ^a^	0.50 ± 0.051 ^a^	T1. 358 T2. 397 T3. 442	−12	2.75 ± 0.14 ^ab^	244.03 ± 33.40 ^ab^
PU_ExtB	78.84 ± 7.18 ^ab^	0.49 ± 0.076 ^a^	(T. 296) T1. 353 T2. 393 T3. 434	−8.9	1.86 ± 0.15 ^b^	210.49 ± 24.81 ^abc^
PUCD_ExtB	79.47 ± 3.78 ^ab^	0.46 ± 0.097 ^a^	T1. 357 T2. 400 T3. 432	−14	2.03 ± 0.31 ^b^	217.17 ± 37.22 ^abc^
PU_ICB	81.48 ± 3.92 ^a^	0.39 ± 0.044 ^a^	T1. 352 T2. 396 T3. 430	−15.8	2.53 ± 0.22 ^ab^	226.07 ± 44.85 ^ab^
PU_ExtS	62.31 ± 5.32 ^b^	0.52 ± 0.044 ^a^	(T. 305) T1. 356 T2. 400 T3. 435	−16.9	2.73 ± 0.58 ^ab^	164.26 ± 22.81 ^bc^
PUCD_ExtS	62.01 ± 5.11 ^b^	0.18 ± 0.028 ^b^	(T. 310) T1. 357 T2. 396 T3. 435	−14.5	2.97 ± 0.29 ^a^	130.27 ± 9.56 ^c^
PU_ICS	83.02 ± 3.55 ^a^	0.50 ± 0.043 ^a^	T1. 356 T2. 395 T3. 436	−15.9	3.23 ± 0.29 ^a^	210.76 ± 41.84 ^abc^

* Nonparametric statistical analysis (Kruskal–Wallis and Dunn comparison test), samples with the same letter do not have a significant difference. ** Parametric statistical analysis (ANOVA and Tukey comparison test), samples with the same letter do not have a significant difference. ^1^ Values are reported as mean ± standard deviation (*n* = 10). ^2^ Values are reported as mean ± standard deviation (*n* = 3).

**Table 3:** Blood compatibility assays and HDFa cell viability of the polyurethane films.

PU	Hemolysis (%) *^1^	Clot (ABS) t = 10 min *^1^	Clot (ABS) t = 20 min	Clot (ABS) t = 40 min	Platelet Adhesion (ABS) *^1^	Viability (%) *^1^
Control	100	0.27 ± 0.0025 ^b^	0.069 ± 0.0026 ^a^	0.068 ± 0.0066 ^a^	0.29 ± 0.077 ^a^	DMSO ^2^
PU	0.70 ± 0.16 ^abc^	0.051 ± 0.0090 ^a^	0.083 ± 0.033 ^a^	0.053 ± 0.0085 ^a^	0.28 ± 0.016 ^a^	93.33 ± 5.74 ^a^
PUCD	0.83 ± 0.098 ^ab^	0.052 ± 0.0062 ^a^	0.048 ± 0.0062 ^a^	0.074 ± 0.016 ^a^	0.26 ± 0.062 ^a^	74.67 ± 3.64 ^b^
PU_ExtB	0.77 ± 0.26 ^abc^	0.062 ± 0.0051 ^a^	0.065 ± 0.0047 ^a^	0.078 ± 0.017 ^a^	0.29 ± 0.005 ^a^	51.77 ± 5.20 ^de^
PUCD_ExtB	1.05 ± 0.34 ^a^	0.052 ± 0.0078 ^a^	0.076 ± 0.047 ^a^	0.088 ± 0.058 ^a^	0.29 ± 0.041 ^a^	60.04 ± 1.51 ^cd^
PU_ICB	0.42 ± 0.11 ^bc^	0.050 ± 0.0083 ^a^	0.099 ± 0.043 ^a^	0.062 ± 0.0096 ^a^	0.27 ± 0.011 ^a^	71.82 ± 4.05 ^bc^
PU_ExtS	0.29 ± 0.10 ^c^	0.056 ± 0.0079 ^a^	0.052 ± 0.012 ^a^	0.055 ± 0.0050 ^a^	0.34 ± 0.008 ^a^	37.06 ± 1.62 ^f^
PUCD_ExtS	0.78 ± 0.11 ^abc^	0.048 ± 0.0026 ^a^	0.053 ± 0.0097 ^a^	0.057 ± 0.0015 ^a^	0.31 ± 0.048 ^a^	41.63 ± 1.57 ^ef^
PU_ICS	0.82 ± 0.078 ^ab^	0.054 ± 0.017 ^a^	0.057 ± 0.078 ^a^	0.093 ± 0.034 ^a^	0.32 ± 0.039 ^a^	51.77 ± 6.29 ^de^

* Statistical analysis; samples with the same letter do not have a significant difference. ^1^ Values are reported as mean ± standard deviation (*n* = 3). ^2^ Positive controls: 70.89 ± 5.41 ^bc^ (DMSO 2.5%), 30.39 ± 7.26 ^f^ (DMSO 5%), and 15.15 ± 1.91 ^ef^ (DMSO 10%).

## Data Availability

Data are contained within this article.
